# Clinical Characteristics of the Cervical Inlet Patch: A Case Series

**DOI:** 10.1002/oto2.24

**Published:** 2023-02-17

**Authors:** Julian S. De La Chapa, Christopher J. Harryman, Patrick O. McGarey, James J. Daniero

**Affiliations:** ^1^ Department of Otolaryngology–Head and Neck Surgery University of Virginia Charlottesville Virginia USA

**Keywords:** cervical inlet patch, cricopharyngeal dysfunction, gastric inlet patch, heterotopic gastric mucosa, inlet patch, laryngopharyngeal reflux

## Abstract

**Objective:**

The goal of this study was to characterize the symptoms and outcomes of patients with a symptomatic cervical inlet patch (CIP).

**Study Design:**

Retrospective case series.

**Setting:**

Tertiary care laryngology clinic in Charlottesville, Virginia.

**Methods:**

A retrospective chart review of the patient's demographics, comorbidities, prior workup, interventions, and response to treatment was performed. All patients received flexible nasolaryngoscopy and barium swallow study. The analysis was descriptive.

**Results:**

Eight patients (6 female) were followed for the management of symptoms related to CIP. The mean age at presentation to our clinic was 64.9 (standard deviation = 15.7). Five out of 8 patients presented with a chief complaint of dysphagia, and the remaining 3 with chronic coughs. Five out of 8 patients demonstrated findings of laryngopharyngeal reflux (LPR) including vocal fold edema, mucosal erythema, or postcricoid edema. Swallow study demonstrated hiatal hernia in 3 of 8 patients, and cricopharyngeal (CP) dysfunction (CP hypertrophy, CP bar, and Zenker's diverticulum) in 3 of 8 patients. One patient presented with a history of Barrett's esophagus. Treatment included increased acid suppression therapy and management of coexisting esophageal pathologies. Ablative procedures were performed in 5 out of 8 cases, with 2 patients requiring repeat procedures. All patients experience subjective symptom improvement.

**Conclusion:**

CIP tends to present in complex patients with multifactorial dysphagia, with the most common symptoms being dysphagia and cough. Clinical features of CIP overlap with other more common pathologies encountered by otolaryngologists including LPR and CP dysfunction, and future prospective studies in larger populations should seek to clarify these associations.

The gastric inlet patch, also known as a cervical inlet patch (CIP), is an anatomical anomaly wherein secretory gastric mucosa is present in the esophagus, most commonly in the proximal portion. The prevalence of this condition is disputed in the literature, as it is often overlooked in a clinical setting due to difficulty with visualization on routine endoscopy and unclear clinical relevance.^1^ However, an increasing number of studies have shown that inlet patches may be more clinically relevant than previously thought. Sources have claimed a range of 0.18% to 14% prevalence among the general population.^2^, ^3^, ^4^ The most accepted hypothesis for the pathogenesis of CIP proposes that CIP results from the incomplete transformation from columnar to squamous epithelium during embryogenesis.^4^ This is supported by a study that found glucagon‐secreting cells present in CIP, which are absent in adult gastric tissue and present in the embryonic stomach.^5^

Although many cases of CIP are asymptomatic, symptoms of CIP are thought to result from the secretion of acid from gastric parietal cells within the heterotopic mucosa, which can then reflux into the larynx and pharynx causing cough, globus, dysphagia, and excessive throat clearing.^4^ There is some evidence that the severity of symptoms correlates with CIP size.^6^, ^7^, ^8^ Esophageal narrowing and stricture formation may occur due to chronic inflammation requiring surgical intervention. Other known sequelae include laryngitis, esophagitis, esophageal web, ulcer, perforation, and fistula formation.^9^ Malignant transformation is also a rare but serious risk.^2^, ^4^, ^10^, ^11^ Other conditions associated with CIP include gastroesophageal reflux disease (GERD), laryngopharyngeal reflux (LPR), and Barrett's esophagus.^3^, ^4^, ^7^, ^12^

Diagnosis can be made fluoroscopically or via direct endoscopic visualization. Endoscopically, CIP appears as a flat, velvety, rose or salmon‐colored patch located between 15 and 21 cm from the central incisors.^4^ Patches vary in size from microscopic to as large as circumferential lesions 4 cm in length.^10^ Biopsy most commonly shows the presence of fundic mucosa containing acid‐secreting parietal and chief cells but may also show “transitional” mucosa containing a mixture of fundic and antral glands.^3^, ^13^

Treatment for symptomatic cervical inlet patches typically begins with proton pump inhibitor (PPI) therapy. For cases refractory to medical management or showing evidence of dysplasia or neoplasia, endoscopic argon plasma ablation,^14^ radiofrequency ablation,^15^ or submucosal/mucosal dissection is indicated.^3^ Although less common, the overlap between common causes of reflux, cough, and dysphagia makes CIP a clinically significant entity worthy of investigation. The purpose of this case series was to characterize the symptoms and outcomes of patients with symptomatic CIP.

## Methods

This study design was reviewed and approved by the University of Virginia Institutional Review Board for Health Science Research (IRB‐HSR) prior to data collection (IRB‐HSR # 22341). Written informed consent for patient information and images to be published was provided by the patients or a legally authorized representative. Records of all patients with a diagnosis of cervical inlet patch up to June 2022 were retrospectively identified with our electronic medical record database using a pre‐existing list of patients seen in the clinic with a diagnosis of CIP or ectopic gastric mucosa. All patients from this cohort were seen in the University of Virginia Voice and Swallowing Clinic during this period. Demographic data, flexible nasolaryngoscopy, esophagoscopy, barium swallow studies, and patient reports of symptoms were obtained. Descriptive statistics were used to summarize baseline characteristics.

## Results

Eight patients (6 female) were identified as having ectopic gastric mucosa in the proximal esophagus with a mean age of 64.9 (standard deviation [SD] = 15.7) at the time of initial evaluation. The average body mass index for this cohort was 31.1 (SD = 10.4). Two patients reported intermittent alcohol use, 1 endorsed a 50‐pack‐year smoking history, and the remaining 5 denied any substance use (Table 1). None of the patients presented with a history of noncutaneous neoplasms or prior radiation exposure. Other comorbidities included diabetes (2/8), asthma (2/8), prior cerebrovascular accident (2/8), cerebral palsy, seizure disorder, muscle tension dysphonia, and cervical stenosis with anterior cervical discectomy and fusion (Table 2).

**Table 1:** Patient Demographics and Clinical Characteristics

Patient	Age (y)	Sex	BMI	Ethnicity	Substance history	Pulmonary comorbidity	Diabetes
1	34	F	23.8	Caucasian	None	N	N
2	81	M	23.7	Caucasian	50‐pack year smoker	N	N
3	77	F	24.5	Caucasian	None	N	N
4	65	F	34.5	African American	None	Asthma	Y
5	75	M	22.2	Caucasian	Intermittent alcohol use	N	N
6	56	F	41.9	Caucasian	None	N	Y
7	75	F	26.9	Caucasian	None	N	N
8	56	F	50.7	African American	Intermittent alcohol use	Asthma	N

Abbreviations: BMI, body mass index; F, female; M, male; N, no; Y, yes.

**Table 2:** Patient Demographics and Clinical Characteristics

Patient number		LPR	OSA		GERD	Presence of hernia	Other comorbidities
1		N	N		Y	N	Cerebral palsy
2		Y	N		Y	Hiatal hernia	Cervical stenosis s/p ACDF, muscle tension dysphonia
3		N	N		Y	N	None
4		Y	N		Y	Hiatal hernia	Prior CVA
5		Y	N		Y	Hiatal hernia	Prior CVA
6		N	Y		Y	N	Seizure history
7		Y	N		Y	N	None
8		Y	Y		Y	N	None

Abbreviations: ACDF, anterior cervical disc fusion; CVA, cerebrovascular accident; GERD, gastroesophageal reflux disease; LPR, laryngopharyngeal reflux; N, no; OSA, obstructive sleep apnea; Y, yes.

Five patients presented with a chief complaint of dysphagia and the remaining 3 with chronic coughs. Other symptoms included dysphonia, odynophagia, and globus sensation. Four patients received a diagnosis of cervical inlet patch prior to their presentation to otolaryngology and all patients had been placed on a reflux regimen prior to evaluation by our clinic. Several had multidisciplinary evaluations including pulmonology, allergy, and rhinology.

### Endoscopic Examination and Voice/Swallowing Evaluation

Esophagoscopy performed on each patient provided visualization of the inlet patch in the expected position 15 to 21 cm from the central incisors. Four patients were found to have a single patch, with 3 patients having multifocal patches. The estimated circumference of the patches ranged from <25% to 80%. Biopsies demonstrated either junctional mucosa or gastric epithelium with inflammation with no evidence of malignancy.

A fluoroscopic barium swallow study revealed hiatal hernia in 3 of 8 patients. Cricopharyngeal (CP) dysfunction, including CP hypertrophy and Zenker's diverticulum, was found in 3 out of 8 patients. Killian Jamieson diverticulum was present in 1 patient. Flexible nasolaryngoscopy demonstrated findings of LPR, including vocal fold edema, mucosal erythema, or postcricoid edema in 5 out of 8 patients. A validated scoring system assessing LPR symptoms was not used in these patients, and diagnosis of LPR was based on patient‐reported symptoms and clinical findings.

### Interventions

The treatment strategy began with a high‐dose reflux regimen in 6 out of 8 patients. This included increased PPI dosage and/or frequency, and/or addition of alginate or H2 blocker. In addition to CIP management, 5 patients were also managed for coexisting pathologies of the esophagus. Three patients required esophageal dilation due to CP dysfunction. In 1 patient with CIP and a small Killian‐Jamison diverticulum, CP and upper cervical esophagus botulinum toxin injections were performed. CO_2_ laser diverticulotomy was performed in 1 patient with CIP and Zenker's diverticulum. One patient was previously diagnosed with Barrett's esophagus. Five patients had their CIP addressed surgically with varying techniques including coblation (2), potassium‐titanyl‐phosphate (KTP) laser ablation (1), hot biopsy forceps ablation (1), or all 3 (1). Two patients required repeat ablative procedures. Variation in the technique depended on the ability to achieve adequate rigid exposure for intervention versus a flexible endoscopic approach.

### Outcomes

Symptoms recurred in 3 patients following surgical intervention. Upon repeat endoscopy, 1 patient was found to have no evidence of CIP following ablation. The remaining 2 had repeat procedures, with 1 patient experiencing improvement in symptoms and the other with no subjective improvement (Table 3).

**Table 3:** Outcomes

Patient number	CIP % circumference	Intervention	Subjective patient‐reported symptom change
1	<25%, 1 lesion	Esophageal dilation, CP Botox	Dysphagia improved
2	80%, multifocal	Esophageal dilation, KTP CIP ablation	Dysphagia improved
3	25%‐30%, 1 lesion	Esophageal dilation	Dysphagia improved
4	75%‐80%, multifocal	Electrocautery ablation of CIP	Dysphagia, odynophagia, globus, and cough improved. Heartburn worse
5	33%, 1 lesion	Coblation of CIP	Improvement in positional cough and throat clearing
6	NR	CO_2_ diverticulotomy	Dysphagia improved
7	50%, multifocal	Coblation of CIP	Dysphagia, globus, cough, odynophagia, throat clearing completely resolved
8	>25%, 1 lesion	CIP hot biopsy forceps ablation	Dysphagia and odynophagia improved

Abbreviations: CIP, cervical inlet patch; KTP, potassium‐titanyl‐phosphate; NR, not reported.

## Discussion

Once considered a rare and clinically irrelevant endoscopic finding, an expanding collection of the literature suggests CIP is an underreported and clinically relevant entity capable of contributing to esophageal and extraesophageal symptoms. This case series illustrates the importance of thorough endoscopic and radiographic evaluation of complicated patients presenting with dysphagia and chronic cough refractory to antisecretory medical treatment. The question remains how much the symptoms of this patient population can be directly attributed to CIP; however, the results of this case series suggest that some patients may benefit from ablative treatment of CIP in addition to the treatment of coexisting esophageal pathologies.

### CP Dysfunction

There was a high prevalence of CP dysfunction in this patient population. CP dysfunction, including CP hypertrophy, CP bar, and Zenker's diverticulum is typically managed surgically by esophageal dilation, botulinum toxin injection, or diverticulectomy. In this study, all 4 patients with concomitant CIP and CP dysfunction were managed surgically, with only 1 patient (#2) receiving KTP laser ablation for CIP treatment. In each case, dysphagia improved following the treatment of CP dysfunction. Dysphagia initially improved in patient 2 following dilation and ablation but soon recurred. Upon repeat esophagoscopy and KTP ablation, the patient experienced no improvement in her dysphagia. Of note, this patient also had the largest (80% circumferential, 2 cm in length) patch in this case series, while the other patients with CP dysfunction had smaller (<30%) patches. Further studies should be performed to better characterize the role of treatment of CIP in patients with CP dysfunction, as these 2 pathologies could be acting synergistically in their contribution to dysphagia.

### LPR

Symptoms of patients with CIP overlap with symptoms of LPR, and the association of these conditions is the subject of active debate. Once thought to be the result of the reflux of gastric contents into the laryngopharynx, the association with CIP is challenging this theory. The proximity of the inlet patch to the laryngopharynx and the superficial location of sensory nerves in the proximal esophagus^3^ support the plausibility of CIP contributing significantly to symptomatic LPR. While dual‐probe pH testing was not performed on the patients in this study, all patients experienced symptoms of classic GERD and were placed on antisecretory medications prior to presentation with minimal to no improvement in symptoms. Our study supports the findings of previous studies that have shown a higher prevalence of LPR in patients with heterotopic gastric mucosa in the esophagus.^7^ Of the 5 patients with LPR in our study, all had improvement in their symptoms following the ablation of their CIP. One patient (#7) had complete resolution of symptoms following inlet patch coblation cauterization. This suggests that CIP may be a contributing factor to LPR severity and emphasizes the importance of esophageal evaluation in patients with LPR refractory to medical treatment.

### Size

Patch circumference is related to the severity of symptoms. The 2 patients (#2 and #4) with the largest patches (75%‐80% circumferential) required repeat ablation, suggesting counseling for this population should include management of expectations following initial ablative treatment.

Treatment of patient 2 is ongoing; their symptoms improved after KTP ablation #1, but then recurred with no improvement following repeat KTP ablation. Although the small sample size limited meaningful statistical analysis, there was no apparent relationship between biopsy findings and symptom patterns, severity, or outcomes.

### The Role of a Biopsy in the Management of CIP

All patients in this study received a biopsy of patch mucosa during esophagoscopy, confirming the condition. While definitive conclusions cannot be drawn from this small sample size, there appears to be no association between biopsy findings and symptom severity or response to treatment. Further studies should be carried out to determine if secretory cell types found in inlet patches are associated with an increased incidence of LPR.

### Limitations

Incomplete subjective patient data, including Voice Handicap Index‐10 and Eating Assessment Tool‐10 scores, and a small sample size limited a meaningful statistical analysis in this case series. Due to the high prevalence of reflux in CP dysfunction patients, we cannot conclude whether CP dysfunction is a direct result of CIP or a reflux‐related entity. Additionally, direct endoscopic visualization for patients with improved symptoms after the intervention was not obtained routinely, restricting the correlation of symptom resolution with the visual improvement of inlet patches.

## Conclusions

Clinicians should maintain a high index of suspicion for CIP in patients presenting with dysphagia and/or chronic cough refractory to antisecretory treatment. There is a high prevalence of LPR in this patient population. Ablative procedures may improve symptoms in patients with CIP refractory to medical treatment. Patients with CP dysfunction may have concomitant CIP. The management of these patients can be complicated, and further studies are required to better characterize this relationship and assess the role of ablative procedures in this population.

## Author Contributions

**Julian S. De La Chapa**, assisted with study design, data collection, data analysis, and manuscript preparation; **Christopher J. Harryman**, assisted with study design, data collection, data analysis, and manuscript preparation; **Patrick O. McGarey**, assisted with study design, data analysis, and manuscript preparation; **James J. Daniero**, assisted with study design, analysis, and manuscript preparation

## Disclosures

### Competing interests

None.

### Funding source

None.

## References

[1] Maconi G , Pace F , Vago L , Carsana L , Bargiggia S , Porro GB . Prevalence and clinical features of heterotopic gastric mucosa in the upper oesophagus (inlet patch). Eur J Gastroenterol Hepatol. 2000;12(7):745‐749. 10.1097/00042737-200012070-00005 10929900

[2] Leshchinskiy S , Ali N , D'Agostino R . The inlet patch. Abdom Radiol. 2018;43(10):2882‐2884. 10.1007/s00261-018-1532-1 29508009

[3] Cock C , Hamarneh Z . Gastric inlet patches: symptomatic or silent. Curr Opin Otolaryngol Head Neck Surg. 2019;27(6):453‐462. 10.1097/MOO.0000000000000581 31567494

[4] Rusu R , Ishaq S , Wong T , Dunn JM . Cervical inlet patch: new insights into diagnosis and endoscopic therapy. Frontline Gastroenterol. 2018;9(3):214‐220. 10.1136/flgastro-2017-100855 30046427 PMC6056090

[5] Feurle GE , Helmstaedter V , Buehring A , Bettendorf U , Eckardt VF . Distinct immunohistochemical findings in columnar epithelium of esophageal inlet patch and of Barrett's esophagus. Dig Dis Sci. 1990;35(1):86‐92. 10.1007/BF01537228 2295298

[6] Baudet JS , Alarcón‐Fernández O , Sánchez Del Río A , Aguirre‐Jaime A , León‐Gómez N . Heterotopic gastric mucosa: a significant clinical entity. Scand J Gastroenterol. 2006;41(12):1398‐1404. 10.1080/00365520600763094 17101570

[7] Chong VH , Jalihal A . Heterotopic gastric mucosal patch of the esophagus is associated with higher prevalence of laryngopharyngeal reflux symptoms. Eur Arch Otrhinolaryngol. 2010;267(11):1793‐1799. 10.1007/s00405-010-1259-2 20437050

[8] Poyrazoglu OK , Bahcecioglu IH , Daglı AF , Ataseven H , Celebi S , Yalniz M . Heterotopic gastric mucosa (inlet patch): endoscopic prevalence, histopathological, demographical and clinical characteristics. Int J Clin Pract. 2009;63(2):287‐291. 10.1111/j.1742-1241.2006.01215.x 17535303

[9] Peitz U , Vieth M , Evert M , Arand J , Roessner A , Malfertheiner P . The prevalence of gastric heterotopia of the proximal esophagus is underestimated, but preneoplasia is rare—correlation with Barrett's esophagus. BMC Gastroenterol. 2017;17(1):87. 10.1186/s12876-017-0644-3 28701149 PMC5508702

[10] Orosey M , Amin M , Cappell MS . A 14‐year study of 398 esophageal adenocarcinomas diagnosed among 156,256 EGDs performed at two large hospitals: an inlet patch is proposed as a significant risk factor for proximal esophageal adenocarcinoma. Dig Dis Sci. 2018;63(2):452‐465. 10.1007/s10620-017-4878-2 29249048

[11] Basseri B , Conklin JL , Mertens RB , Lo SK , Bellack GS , Shaye OA . Heterotopic gastric mucosa (inlet patch) in a patient with laryngopharyngeal reflux (LPR) and laryngeal carcinoma: a case report and review of literature. Dis Esophagus. 2009;22(4):E1‐E5. 10.1111/j.1442-2050.2008.00915.x 19473208

[12] Avidan B , Sonnenberg A , Chejfec G , Schnell TG , Sontag SJ . Is there a link between cervical inlet patch and Barrett's esophagus. Gastrointest Endosc. 2001;53(7):717‐721. 10.1067/mge.2001.114782 11375577

[13] Jabbari M , Goresky CA , Lough J , Yaffe C , Daly D , Côté C . The inlet patch: heterotopic gastric mucosa in the upper esophagus. Gastroenterology. 1985;89(2):352‐356. 10.1016/0016-5085(85)90336-1 4007426

[14] Bajbouj M , Becker V , Eckel F , et al. Argon plasma coagulation of cervical heterotopic gastric mucosa as an alternative treatment for globus sensations. Gastroenterology. 2009;137(2):440‐444. 10.1053/j.gastro.2009.04.053 19410576

[15] Dunn JM , Sui G , Anggiansah A , Wong T . Radiofrequency ablation of symptomatic cervical inlet patch using a through‐the‐scope device: a pilot study. Gastrointest Endosc. 2016;84(6):1022‐1026. 10.1016/j.gie.2016.06.037 27373671

